# The microbiome seeding debate – let’s frame it around women-centred care

**DOI:** 10.1186/s12978-019-0747-0

**Published:** 2019-06-28

**Authors:** A.U. Lokugamage, S.D.C. Pathberiya

**Affiliations:** 1 International MotherBaby Childbirth Organisation (NGO), Ponte Vedra Beach, Florida USA; 2 Department of Women’s Health, Whittington Hospital, London, UK; 3 University College London Medical School, London, UK; 4 Acculegal Solicitors, London, UK

**Keywords:** Microbiome seeding, Caesarean section, Ecology, Feminism, Non-communicable diseases

## Abstract

**Abstract:**

In a global culture that is increasingly interested in ecological interventions, probiotics, ‘friendly bacteria’, microbiome preservation/restoration and long-term health, there is growing awareness of the idea of seeding the vaginal microbiome in the new born after caesarean section. It is postulated as a way of restoring helpful missing microbes and preventing long term non-communicable diseases of babies delivered by caesarean section. Currently, there is a deluge of evidence being published on the human microbiome, which can be challenging to digest and absorb by scientists, clinicians and patients. The specific evidence base around this technique is at its early stages. This commentary discusses what advice is currently available from a feminist and a person-centred care perspective.

**Abstrakt:**

Det er en voksende interesse internasjonalt for økologiske intervensjoner, probiotika, ‘snille bakterier’, bevaring/gjenoppretting av. mikrobiomet og helse i et langtidsperspektiv. I denne sammenhengen er det en økende interesse for tanken om å så det vaginale mikrobiomet (vaginal seeding) på den nyfødte etter et keisersnitt. Dette er postulert som en måte å gjenopprette manglende normalflora/mikrobiom og forebygge langvarige ikke-smittsomme sykdommer hos barn født med keisersnitt. For tiden publiseres det mye forskning om menneskets mikrobiom, noe som kan være utfordrende å fordøye og ta til seg for forskere, klinikere og pasienter. Forskningen på denne spesifikke metoden er i sin begynnelse. Denne kommentaren drøfter hvilke råd som for øyeblikket er tilgjengelige, fra et feministisk og personsentrert omsorgsperspektiv.

**Popularisert sammendrag på norsk:**

Det menneskelige mikrobiomet er summen av alle bakteriene som dekker den menneskelige kroppen og det hjelper kroppen i å fungere optimalt. Når mikrobiomet forstyrres, vil kroppen kunne få betennelsesreaksjoner og allergier. I fødsel finnes de «gode» bakteriene i kvinnens vagina. (det vaginale mikrobiomet) som man tror vil være fordelaktig for babyens evne til å utvikle et sunt immunsystem. Babyer som er født med keisersnitt vil ikke bli eksponert for disse «gode» bakteriene og det kan påvirke barnets immunforsvar negativt og potensielt øke sjansen for allergier og betennelsesreaksjoner i kroppen på lang sikt. Vaginal seeding (et forsøk på å gjenopprette balansen og noen av de gode bakterier i spedbarnet gjennom å tilføre mors vaginale bakterier via en kompress som strykes over spedbarnets ansikt) Vaginal seeding er en metode som noen forskere sier muligens delvis gjenoppretter de manglende «gode» bakteriene etter et keisersnitt. Forskningen er på et tidlig stadium. Det har vært avisartikler og en film om emnet og mødre har funnet ut om vaginal seeding som en måte å gjenopprette denne delen av babyens mikrobiom. Foreldre ønsker å diskutere vaginal seeding, men på nåværende tidspunkt er helsevesenet avventende og helsepersonell er ikke godt nok informert. Denne artikkelen vil se på den pågående diskusjonen.

**Résumé:**

Dans une culture mondiale qui s’intéresse de plus en plus aux interventions écologiques, aux probiotiques, aux «bactéries amicales», à la préservation / restauration du microbiome et à la santé à long terme, on commence à prendre conscience de l’idée d’ensemencer le microbiome vaginal chez le nouveau-né après une césarienne. Il est postulé comme un moyen de restaurer les microbes manquants et d'aider à prévenir les maladies non transmissibles à long terme des bébés mis au monde par césarienne. Il existe actuellement un déluge de preuves sur le microbiome humain, qui peuvent être difficiles à digérer et à absorber par les scientifiques, les cliniciens et les patients. La base de preuves spécifique autour de cette technique en est à ses débuts. Ce commentaire discute des conseils actuellement disponibles dans une perspective de soins féministe et centrée sur la personne.

**Résumé simplifié:**

Le microbiome humain est constitué de tous les microbes qui recouvrent le corps humain et qui aident le corps à bien fonctionner. Lorsque le microbiome est perturbé, le corps devient plus inflammatoire et est sujet aux allergies. Lors de l’accouchement, le vagin d’une mère (le microbiome vaginal) contient des "bactéries amicales" qui pourraient être bénéfiques pour l’enfant et aider le bébé à développer un système immunitaire en bonne santé. Les bébés nés par césarienne ne sont généralement pas exposés à ces «bactéries bénéfiques», ce qui pourrait affecter négativement le système immunitaire du bébé et potentiellement augmenter le risque d’allergies et d’inflammation à long terme. Selon certains scientifiques, l'ensemencement vaginal pourrait partiellement restaurer les «bactéries amies» manquantes après la césarienne. La recherche en est à ses débuts. Il y a eu des articles de journaux et un film à ce sujet, et les mères ont découvert l'existence d'un ensemencement vaginal (où une compresse placée dans le vagin de la mère pourrait être appliquée sur l'enfant après la césarienne) afin de restaurer une partie du microbiome du bébé. Les parents souhaitent discuter de l'ensemencement vaginal, mais à l'heure actuelle, les organisations médicales sont prudentes et les praticiens ne sont pas suffisamment informés. Cet article examine le débat en cours.

**RESUMO:**

Numa cultura global que está cada vez mais interessada em intervenções ecológicas, probióticos, “bactérias amigáveis”, preservação/restauração do microbioma e saúde a longo prazo, há uma crescente consciência sobre a ideia de semear o microbioma vaginal no recém-nascido após uma cirurgia cesariana. Isso está sendo postulado como uma forma de restaurar micróbios úteis que lhe faltariam e prevenir doenças não transmissíveis em longo prazo para bebês que nasceram pela via cirúrgica. Atualmente, há um aumento massivo de evidências sendo publicadas sobre o microbioma humano cuja absorção e digestão pode ser desafiadora para cientistas, clínicos e pacientes. A base específica da evidência que cerca essa técnica ainda está em estágios preliminares. Este comentário discute o aconselhamento atualmente disponível numa perspectiva feminista e centrada na pessoa.

**Síntese simplificada:**

O microbioma humano está composto por todos os micróbios que cobrem o corpo humano e que ajudam o corpo a funcionar bem. Quando o microbioma é perturbado, o corpo tem mais inflamações e maior propensão a desenvolver alergias. Ao nascimento, há “bactérias amigáveis” na vagina materna (o microbioma vaginal) que podem ser benéficas à criança e ajudar o bebê a desenvolver um sistema imunológico saudável. Bebês que nascem por cesariana usualmente não são expostos a essas “bactérias amigáveis” e isso poderá afetar negativamente o sistema imunológico do bebê, aumentando potencialmente a probabilidade de alegrias e inflamações no longo prazo. A semeadura de bactéria vaginais é um método que alguns cientistas afirmam que poderá restaurar parcialmente as “bactérias amigáveis” faltantes depois de uma cesariana. Essa pesquisa está em fase preliminar. Houve alguns artigos em jornais e um filme sobre isso, e as mães descobriram a possibilidade da semeadura vaginal (quando é feito um swab da vagina materna que é esfregado no bebê após a cesárea) para restaurar parte do microbioma do bebê. Pais desejam discutir a semeadura vaginal, mas, no momento, as organizações médicas têm sido cautelosas e os profissionais não estão adequadamente informados. Este artigo aborda o debate em andamento.

**Resumen:**

En una cultura global que está cada vez más interesada en las intervenciones ecológicas, los probióticos, las "bacterias amigables", la conservación/restauración de microbiomas y la salud a largo plazo, hay una creciente conciencia de la idea de sembrar el microbioma vaginal en el recién nacido después de la cesárea. Se postula como una forma de restaurar los microbios útiles faltantes y prevenir las enfermedades no transmisibles a largo plazo de los bebés nacidos por cesárea. Actualmente, se está publicando una gran cantidad de pruebas sobre el microbioma humano, que pueden ser difíciles de digerir y absorber por parte de científicos, clínicos y pacientes. La base de la evidencia específica en torno a esta técnica se encuentra en sus primeras etapas. Este artículo analiza qué consejos están disponibles actualmente desde una perspectiva feminista y de atención centrada en la persona.

**Resumen en lenguaje sencillo:**

El microbioma humano está hecho de todos las bacterias que cubren el cuerpo humano y que ayudan al cuerpo a funcionar bien. Cuando se altera el microbioma, el cuerpo se inflama más y es propenso a las alergias. En el parto, hay "bacterias amigables" en la vagina de la madre (el microbioma vaginal) que podrían ser beneficiosas para el niño y ayudar al bebé a desarrollar un sistema inmunológico saludable. Los bebés que nacen por cesárea generalmente no se exponen a estas "bacterias amigables" y esto podría afectar negativamente el sistema inmunológico del bebé, lo que podría aumentar la probabilidad de alergias e inflamación a largo plazo. La siembra vaginal es un método que algunos científicos dicen que podría restaurar parcialmente las "bacterias amigables" que faltan después de la cesárea. La investigación se encuentra en sus primeras etapas. Han habido artículos periodísticos y una película sobre esto, y las madres se han enterado de la siembra vaginal (donde se puede frotar el niño con una torunda de la vagina de la madre después de la cesárea) para restaurar la parte del microbioma del bebé. Los padres quieren hablar sobre la siembra vaginal, pero en la actualidad las organizaciones médicas son cautelosas y los profesionales no están informados adecuadamente. Este artículo analiza el debate en curso.

## Plain English summary

The human microbiome is made of all the bugs that cover the human body and that help the body to function well. When the microbiome is disturbed, the body gets more inflammation and is prone to allergies. In childbirth, there are ‘friendly bacteria’ in a mother’s vagina (the vaginal microbiome) which could be beneficial to the child and help the baby develop a healthy immune system. Babies who are born by caesarean section do not usually get exposed to these ‘friendly bacteria’ and this might negatively affect the baby’s immune system, potentially increasing the likelihood of allergies and inflammation in the long term. Vaginal seeding is a method that some scientists say might partially restore the missing ‘friendly bacteria’ after caesarean section. The research is at its early stages. There have been newspaper articles and a film about this and mothers have found out about vaginal seeding (where a swab taken from the mother’s vagina could be rubbed on to the child after caesarean) to restore part of the baby’s microbiome. Parents are wanting to discuss vaginal seeding but at present medical organisations are cautious and practitioners are not adequately informed. This article looks at the ongoing debate.

## Background

Several cohort studies [1–11] have shown that birth by caesarean section can be associated with an increased risk of autoimmune non-communicable diseases and neurodevelopmental problems in offspring when looked at through a life course epidemiological lens. There is considerable debate [12, 13] in the scientific literature and on social media birth groups as to whether differences in the neonatally acquired gut microbiota between caesarean section and vaginally born babies, impact on autoimmunity and are implicated in long term epigenetic changes. There is also debate as to whether vaginal seeding of the microbiome after caesarean could partially restore the abnormalities in microbiota. This has stirred up growing public interest in finding out information about optimising the neonatal gut and skin microbiome for babies born by caesarean section as they bypass the acquisition of the maternal vaginal and gut microbiota encountered through the vaginal birth route. This is an area of complexity and uncertainty with a juxtaposition between medicine and ecological science. Thus, health care professionals need to learn to navigate this territory with a person-centred care philosophy.

The UK’s General Medical Council (GMC) 2018 [14] guidance for desired professional characteristics of new doctors explicitly states that doctors of the future should be able to negotiate areas of complexity and uncertainty, recognising the complex medical needs, goals and priorities of patients, the factors that can affect a patient’s health and wellbeing and how these interact. The UK Supreme Court in *Montgomery v Lanarkshire *[15] said that the doctor’s duty was *to “.. take reasonable care to ensure that the patient is aware of any material risks involved in the recommended treatment and any reasonable alternative or variant treatments”*. The duty to involve the patient in matters concerning their treatment is also established in international instruments [16, 17]. Doctors should therefore demonstrate working collaboratively with patients, their relatives, carers or other advocates, in planning their care, negotiating and sharing information appropriately and supporting patient self-care. The Supreme Court [15] also said that *“…social and legal developments … point away from a model of the relationship between the doctor and the patient based upon medical paternalism. They also point away from a model based upon a view of the patient as being entirely dependent on information provided by the doctor”.* ^15^ The World Health Organisation’s (WHO) Sustainable Developmental Goals also stress the importance of human rights based care, alongside medical and patient safety issues, which aligns with ethos of respectful maternity care. So, drawing the threads together of dealing with complexity and uncertainty in light of GMC recommendations on professionalism, the UK law and the rights-based style promoted by the WHO, we inquired into the approach taken by opinion articles published on vaginal seeding.

Vaginal seeding is an area that women and their partners have a thirst for information. It follows that empowering women and their partners to make informed decisions about their health, medical treatments and their baby’s long-term health, is an integral part of patient-centred care. The focus of our commentary is on the person-centred or human rights angle in relation to this complex, uncertain and burgeoning area of research.

## Discussion about recently published opinions

There have been a number of medical opinion articles published by the British Medical Journal (BMJ) [18], BJOG, [19, 20] and the American College of Obstetricians and Gynecologists (ACOG) [21] which we have considered in this paper.

In the BMJ [18], Cunnington et al. say that “*We have advised staff at our hospitals not to perform vaginal seeding because we believe the small risk of harm cannot be justified without evidence of benefit. However, the simplicity of vaginal seeding means that mothers can easily do it themselves. Under these circumstances we should respect their autonomy but ensure that they are fully informed about the theoretical risks.”* The ACOG in their Committee Opinion No. 725 [21], *“does not recommend or encourage vaginal seeding outside of the context of an institutional review board-approved research protocol”* however they do not prohibit an initiation of discussion of the subject. A BJOG commentary by Haarh et al. [22] state that they *“do not recommend VS and we further suggest that the discussion of VS should not be initiated by healthcare professionals.”* Eschenbach [20] also in a separate BJOG commentary agrees with the caution of Harrh et al.

These papers discourage the concept of seeding of the microbiome after caesarean section with a vaginal swab due to *potential unknown dangers* and a *paucity of data around the technique.* However, it can be argued that, if the baby had been born vaginally, they would have been exposed to the very same microbes anyway. This side of the debate was not brought to the fore in weighing up the risks. By saying that exposure to vaginal secretions and microbiota could be putting all babies at risk of infection infers that babies born by physiological vaginal birth (the evolutionary norm) is to put babies at risk, because the vaginal contents are potentially dangerous. To unintentionally infer that the average human vagina is dangerous could provoke feminist consternation.

### Why is there public interest?

The public appetite for information in this area may have been piqued by the documentary Microbirth (2014) [23] in which the work of Professor Rodney Dietert, Cornell University, raises the idea of the microbiome and human genome comprising a superorganism which is referred to as the Completed Self. Professor Dietert argues this superorganism cannot be created if there are missing steps vital to create a homeostatic immune system, such as when a neonate does not have an opportunity to have the maternal vaginal microbiome seeded at the time of birth.

The *incomplete-self* thus created leads to misregulated “*inflammation, a host defence-homeostasis disorder* [and] *appears to be a key biomarker connecting a majority of chronic diseases.”* [24] Caesarean sections were designed to save lives but the increase in global caesarean section rates may have unintentional long term consequences. “*We have run the risk of losing certain distinct advantages that were inherently embedded in ancient cultures and practices. Among these were the microbial-rich experiences of natural childbirth, breastfeeding, and agrarian living.”* [25]

In a report on the impending burden of chronic non-communicable diseases (NCD) by the World Economic Forum and Harvard School of Public Health, the following points were made [26].

- NCDs already pose a substantial economic burden and this burden will evolve into a staggering one over the next two decades.
- Cardiovascular disease, chronic respiratory disease, cancer, diabetes and mental health, the macroeconomic simulations suggest a cumulative output loss of US $47 trillion over the next two decades.
- This loss represents 75% of global GDP in 2010 (US$ 63 trillion).

Therefore, from a public health and economic perspective, there is a real interest to look at factors that cause chronic disease in humans. Could microbiomic disturbance have a considerable role to play? Hence public interest in the area of birth and the microbiome may be justified and should not be dismissed.

The documentary also highlights the work of Professor Martin Blaser, Professor of Translational Medicine, Director of the NYU Human Microbiome Program who wrote the book ‘The Missing Microbes’ [27] and his co-worker, Associate Professor Gloria Dominguez Bello, whose work in the basic sciences of the microbiome elegantly mapped the differences in maternal and neonatal gut microbiome between elective caesarean delivery and vaginal birth, in the paper ‘Delivery mode shapes the acquisition and structure of the initial microbiota across multiple body habitats in newborns’ [28]. Professor Phillip Steer and Professor Neena Modi, from the UK, also appeared in the documentary discussing the risks of caesarean sections and long-term non-communicable diseases of the offspring. However, this documentary alluded to the Dominguez Bello group’s research on restoration of the neonatal microbiome after caesarean by using a maternal vaginal swab to seed the vaginal microbiome in the baby by wiping the neonatal body with the vaginally inoculated swab. This work was eventually published as a pilot study in 2016 [29]. Women rapidly caught hold of this message even before the publication of this research [30] and are now asking obstetricians whether they can seed their baby’s microbiome with a vaginal swab but the average obstetrician does not know what to do and advise about this. Further to the opinions of Haarh et al.^22^ and Eschenbach [20] above, any embargo on initiating discussion of this area should be discouraged and health care professionals should develop the language and knowledge to navigate the discussion with parents. Professional societies should provide regularly updated syntheses of evidence to enable informed conversation.

The public should not be too dismayed if there are no current opportunities for attempting to seed the vaginal microbiome on their babies because early skin to skin and exclusive breastfeeding may also help. Neu et al., having looked at the data from three studies, reveal that the likelihood of developing auto immune conditions after caesarean section varies from odds ratios of 1.24 to 1.83 - not making it a complete certainty [31]. However, an alternative argument is made by Stinson et al. who while accepting an association between caesarean delivery with alterations in infant microbiome, suggest that the *“lack of exposure to vaginal microbiota is unlikely to be a major contributing factor” *[12] in the *“increased rate of asthma, allergies, autoimmune disorders and obesity”* and may be a result of other factors.

Moreover, extending the level of complexity, it is uncertain how the hormonal triggers of the onset of labour, the physiological ‘stress’ of labour and vaginal delivery, or the interaction between the composition (other than the microbiome) of amniotic fluid and vaginal secretions contribute to the eventual ‘autoimmune’ status of the baby. Thus, vaginal seeding can only transfer vaginal bacteria to the neonate and cannot substitute for any of these or other unknown factors which may or may not be important in mediating the possible differences in longer term outcomes between vaginally-delivered and caesarean-delivered infants.

### Need for thorough examination of emerging evidence

If one conducts a simple PubMed search on the term *“human microbiome”,* e.g. at the time of writing this commentary yielded 44,000 publications and a year prior to this, the search term yielded 22,000 publications. Therefore, we are dealing with a deluge of new information, most of which is published in non-obstetric journals which is challenging to cognitively process in a short amount of time [32]. So, it is understandable that there has been a translational delay in obstetricians and gynaecologists being made aware of this emerging ecological science.

Large cohort studies have noted an association between caesarean section and autoimmune conditions and some of which are summarised in *Table 1* of the open access paper *‘Dysbiosis in Children Born by Caesarean Section’* by Garcia et al. in July 2018 [33]. Also there are at least 5 prospective studies [34, 35, 36, 37, 38] on the mode of delivery or use of intrapartum antibiotics and paediatric dysbiosis.

There is constant media attention on global ecological issues and there is a sector of the public who aspire to health, agricultural and industrial practices that preserve ecology. With this tide of interest sits the needs of some parents wanting to engage with the ecological concepts of restoration of the microbiome when they perceive it to be distorted. Indeed we know that in *Clostridium difficile* infections after antibiotics one plausible restorative treatment is faecal transplantion [39] or probiotic administration [40]. There is emerging evidence that probiotic foods such as fermented foods and Kefir might be therapeutic adjunctive treatment to diseases which are impacted by gut microbiota disturbances such as mental health [41] and type 2 diabetes [42]. There is a Cochrane review of promising prospective data regarding probiotics to prevent gestational diabetes [43]. There is also evidence that after short term antibiotic use, although the diversity of the microbiota subsequently recovers to resemble the pre-treatment states, the microbiota remained perturbed in some cases for up to 4 years post treatment [44].

To contextualise the importance of the microbiome at birth one must integrate information from many diverse sources which are not necessarily obstetric. Consequently, busy clinicians can suffer cognitive overload from the sheer amount of paperwork that needs to be digested to maintain practice knowledge. Thus, there should be a deep analysis of literature by obstetric societies triangulating all this information, examining the pros and cons of restoration of the microbiome. This could enable the patient to decide on the type of management they would like to engage, which supports giving information that the patient considers to be relevant in a manner the patient can understand [15]. Hence there cannot be a restriction on discussion or an omission of material information that the patients will find important.

There is a need for thorough analysis and triangulation of evidence in this field and its incorporation into guidance for the benefit of professionals, women and their partners. We feel this guidance should transparently examine the following:

- The microbiomic disturbance of the neonatal gut flora after caesarean section
- The use of antibiotics in the first year of life [45, 46]
- The early programming of the immune system at birth [47].
- Group B streptococcus antibiotic prophylaxis and its long-term sequelae [36].
- Research on the ‘Hygiene hypothesis’ papers and caesarean section link to autoimmune conditions
- Microbiome links to mental health, diabetes, obesity [9], inflammatory bowel disease [48] and cancer treatments [49, 50].

### Risks of seeding

In discussing the risk of vaginal seeding, Huynh et al. [51] in December 2018 reported a case of neonatal herpes simplex infection following a vaginal seeding post caesarean section, which does not mention whether infection screening was done prior to the attempted seeding. In reducing the risks associated with vaginal seeding, Dominguez Bello et al’s ongoing studies [29] employ screening for pathogens [HIV,HSV,GBS] prior to vaginal seeding. Moreover, it should be noted that this screening opportunity may not have been available had the child been born vaginally, depending on the policies of individual units. Also, consideration should be given to the fact that there can be a negative screening but there could potentially be a newly acquired infection between screening and birth.

### Evidence behind guidelines and discrepancies in attitudes to interventions

An analysis of the quality of scientific evidence underlying the Royal College of Obstetricians and Gynaecologists Obstetric guidelines (the ‘Green-top Guidelines’) has been conducted [52]. With regard to the guidelines published from December 2007 onwards, the researchers discovered that 40% of all recommendations were based only on Clinical experience (ungraded √); 26% of all recommendations were evidenced by studies rated 2++ (graded D); 16% of all recommendation of best practice was supported by Studies rated 2+ and directly applicable to the targeted population with consistency of results and extrapolated evidence from studies rated 2++(graded C); and the recommendations supported with the highest rated evidence graded B & A accounted for 10% & 8% respectively [52]. Therefore, more than 70% of recommendations had very little scientific evidence behind them. Similar findings were made by analysing other international institutional guidelines [53, 54]. Given that on many occasions’ practices are adopted with lower grades of evidence, parents may feel that the evidence thresholds that are deemed to have been met prior to the adoption of a procedure are arbitrary or subject to bias. They may see the present cautiousness to discuss vaginal seeding as prejudicial to their baby’s health.

### Feminist, equality and human rights issues

As this article’s focus is more on professional behaviours, we have already pointed out that there is a medical cultural issue in which some medical interventions have been introduced on a wide scale without high quality evidence, yet an ecological intervention such as the potential partial restoration of microbiota or the normalisation of non-physiological birth is actively discouraged. It seems that the value of some interventions are considered more than others in the face of low evidence or to quote George Orwell *“All animals are equal, but some animals are more equal than others.” *[55] This could demonstrate that a certain unconscious bias exists in the values and cultures of obstetrics and gynaecological organisations in engaging with the ecological science of the microbiome. Indeed, the GMC’s guidance [14] on professional behaviours for new doctors is to *“recognise the potential impact of their attitudes, values, beliefs, perceptions and personal biases (which may be unconscious) on individuals and groups.”* Therefore, it is valuable to reflect on this when considering the responses of the obstetric community.

Unconsciousness-biases can limit women’s choices and affect concepts of women’s bodily integrity. Bodily integrity is an important feminist concept over the course of the history of childbirth. Obstetrics journals traditionally do not have many materials on the discourse that the history of obstetrics has been linked to the concept of appropriation over women’s bodies [56–59]. Female empowerment is linked to a sense of choice and respect for bodily integrity. Hence a failure of professional bodies to recognise or discuss areas that are important to mothers and their families could compromise respectful maternity care. The discussion about microbiome and birth, and inferences that the vagina and its secretions as dangerous can be seen as a feminist issue.

## Conclusion

We are not saying that an intervention like seeding of the vaginal microbiome should be adopted wholesale without reasonable evidence as clearly this intervention needs further evaluation - studies are ongoing. However, we are highlighting that the current professional stance is inconsistent with attitudes of the public to interventions on which evidence is low. This discrepancy undermines patients’ rights to access information that they feel is very important to them, thus hampering principles of respectful maternity care.

The following practice points are recommended:

1. When discussing the risk of caesarean section, the long-term risk of auto-immunity could be discussed in the consenting process of the caesarean section along with other short-term risks such as infection, bleeding, thromboembolism, damage to adjacent viscera and laceration of the baby. Also, medical professionals should have the capability, knowledge and language to discuss the current evidence around caesarean section and its impact on the neonatal gut microbiota and the effect on intermediate and long-term immunity.
2. There cannot be an embargo on discussing information about vaginal seeding, irrespective of who initiates the conversation.
3. Until there is a reasonable evidence base supporting the vagina seeding technique, institutions are not obliged to offer the service. However, they must be prepared to discuss the evidence around it. Including that *under experimental conditions partial restoration of the microbiota was achieved by vaginal seeding in a very small study where researchers had screened the mothers’ vagina to avoid transmitting infections.*
4. Healthcare professionals and institutions should not directly or indirectly obstruct parents from vaginal seeding if they wish to do it themselves because it is a ‘low-tech’ intervention, but the caveat is that the patient will need to take responsibility for their actions, if the institution does not offer it.
5. Local resource implications would have to be considered as to how parents could access pre-birth vaginal infection screening if desired by parents interested in vaginal seeding.

Consideration of these points enables information offered to be more women-centred, giving helpful safety advice for parents who want to seed the microbiome themselves in their babies.

It should also be mentioned that future studies may elucidate, in a more nuanced way, whether the vaginal microbiome or maternal gut microbiome or probiotics turn out to be the optimal way of seeding the neonatal gut of babies born by caesarean or exposure to intrapartum antibiotics. Future guidelines need a thorough evaluation of the emerging science of the microbiome and the mode of delivery and life course epidemiological links to long-term disease/wellbeing and they should be human rights based.
